# Efficacy of Installation of Temporary Bathing Transfer Aids by Older Adults

**DOI:** 10.1177/23337214241237119

**Published:** 2024-03-13

**Authors:** Megan Rand, James Pelchat, Iris C. Levine, Roger E. Montgomery, Rebecca M. Greene, Emily C. King, Steven M. Pong, Alison C. Novak

**Affiliations:** 1 University of Toronto, ON, Canada; 2 KITE Reseach Institute-University Health Network, Toronto, ON, Canada; 3 VHA Home HealthCare, Toronto, ON, Canada; 4 Carleton University, Ottawa, ON, Canada

**Keywords:** bathing, assistive devices, aging-in-place

## Abstract

Grab bars facilitate bathing and reduce the risk of falls during bathing. Suction cup handholds and rim-mounted tub rails are an alternative to grab bars. The objective of this study was to determine whether older adults could install handholds and tub rails effectively to support bathing transfers. Participants installed rim-mounted tub rails and suction cup handholds in a simulated bathroom environment. Installation location and mechanical loading performance were evaluated. Participant perceptions during device installation and a bathing transfer were characterized. While 85% of suction cup handholds met loading requirements, more than half of participants installed the suction cup handhold in an unexpected location based on existing guidance documents. No rim-mounted tub rails were successfully installed. Participants were confident that the devices had been installed effectively. Suction cup handholds and rim mounted tub rails are easy to install, but clients may need additional guidance regarding where, and how to install them.

## Introduction

Bathing is one of the highest risk at-home activities for older adults (Carter et al., 1997; King & Novak, 2017; Stevens et al., 2011) and bathing-related disability is associated with requirement of home care services (LaPlante et al., 2002). Clinicians, such as occupational therapists, play a critical role in aging-in-place strategies through evaluating and adapting the physical environment (Chippendale & Bear-Lehman, 2010). To mitigate fall risk when entering and exiting a bathtub, the installation of bathroom grab bars is a common strategy recommended by occupational therapists and other clinicians who evaluate the home environment of older adults.

Long-term recommendations to improve bathing safety commonly involve installation of permanent grab bars. There is growing evidence that grab bars are effective for bathing mobility and fall prevention (Aminzadeh et al., 2001; King & Novak, 2017; Levine et al., 2023; Sattin et al., 1998; Sekiguchi et al., 2017, 2020). However, barriers exist to the installation and use of permanent grab bars, such as cost (Statistics Canada, 2017), existing configuration and support structures within the bathroom (Bunn et al., 2008; King et al., 2018), inability to make changes to a rented home (Aminzadeh et al., 2001; Bunn et al., 2008; King et al., 2018), and fear of stigmatization (Aminzadeh et al., 2001; King et al., 2018). Suction cup handholds or rim-mounted tub rails are commonly used as temporary, low-cost alternatives. Temporary handholds and tub rails can be installed quickly, do not require permanent renovations, and can be easily removed or relocated to a different bathroom. However, not all bathing assistive devices offer equal support during a transfer (Batista et al., 2016; Greene et al., 2023; Guay, Vinet et al., 2020; Guitard et al., 2011; King & Novak, 2017; Nirmalanathan, 2019), highlighting the need to evaluate whether temporary handholds and tub rails are effective “out of the box” assistive devices for older adults, or, whether additional clinical or product guidance is required.

Specific features of the design of these devices may affect how easily they can be installed. Knobs, levers, or locking collars may require considerable upper extremity strength and dexterity to manipulate. In addition to physical challenges, the manufacturer instructions may be worded unclearly, contain confusing diagrams, and are sometimes printed in small font sizes or poorly contrasting colors. Some suction cup handholds feature a color indicator when the suction is sufficient to support bathing tasks, however it is unclear how well this feature works, and whether it is easy to use. These challenges contribute to the potential for ineffective installation, and increased risk of falls during bathing activities.

While commercially available handholds and tub rails are marketed to older adults, it is unknown whether such devices can be installed effectively by these intended users. Therefore, we aimed to determine whether older adults could install temporary suction cup handholds and rim-mounted tub rails to support a bathing transfer. We also aimed to characterize perceptions of older adults when installing temporary handholds and tub rails.

## Methods

For this study, we followed a mixed-methods approach, employing quantitative and qualitative usability testing with human participants, and quantitative materials testing of the assistive devices as installed by the participants.

### Participants

A convenience sample was recruited via newspaper ads and posters. By telephone screening we determined if participants met the study inclusion criteria. Participants were included if they were: (1) over the age of 65; (2) English speaking; (3) able to attend an in-person session in our laboratory; (4) able to independently step into and out of the bathtub; and/or if they (5) did not recently experience an acute hand/wrist injury. Participants were instructed to bring reading glasses to their study appointment, if they required them.

### Devices

**Figure 1.**
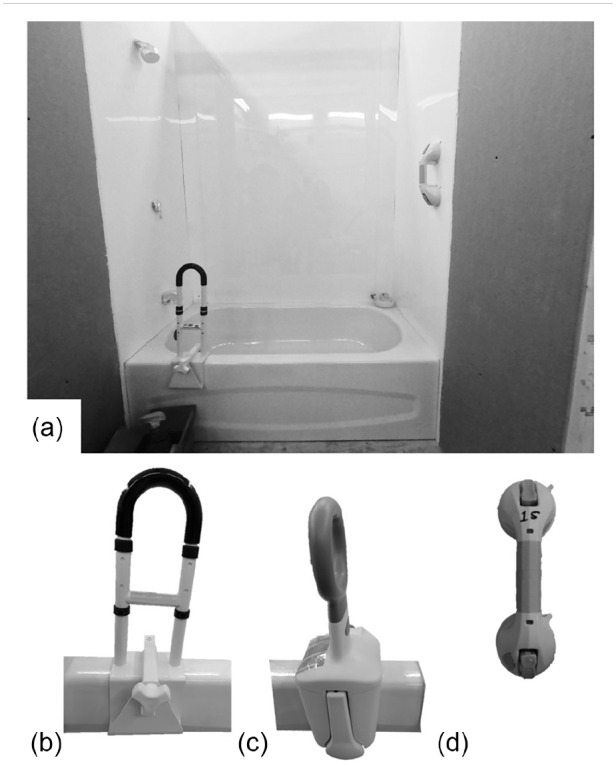
The simulated bathtub environment: (a) the experimental environment, showing the bathtub, bathtub walls, unfinished wall material outside the bathtub area, and the box of study materials (unscented cleaning spray visible). An example finished installation of one Suction Cup Handhold and one Rim-Mounted Tub Rail are shown, (b) Rim-Mounted Tub Rail A, (c) Rim-Mounted Tub Rail B, and (d) Suction Cup Handhold. Identifying marks on the products have been blurred.

Following an environmental scan of commercially available products, two rim-mounted tub rails and one suction cup handhold were selected (Figure 1). All products reviewed were available in Canada from at least two sources, and were indicated by the manufacturer as products to support bathing transfers. Many of the products in our environmental scan were of similar design, with minor esthetic differences, such as color. We did not evaluate quality or effectiveness prior to use in the study. None of the devices were regulated under any existing requirements for effectiveness (e.g., load tolerance). While some of the devices included claims to meeting “ADA requirements,” the exact specifications were not identified. The Suction Cup Handhold featured two rubber disks attached to springs, with a plastic lever to control each spring. To install the Suction Cup Handhold, the manual instructs the user to clean the suction disks and attachment surface, press the disks to the surface, then press down the lever to latch the device. Upon installation, indicators along the handle change from red to green to indicate that a “safe and secure” attachment has been achieved. The user is instructed to test whether the device is secure before each use, but no specific instructions about how to do this test are given. Rim-Mounted Tub Rail A was constructed from white powder-coated steel. Rim-Mounted Tub Rail A was characterized by a knob adjustable rubber-lined, pressure-fit clamp, and featured a height-adjustment control consisting of telescoping tube with locking pins. To install Rim-Mounted Tub Rail A, the user is instructed to clean and dry the bathtub, use the knob to adjust the width of the clamp, and tighten until secure. The user is instructed to test the installation of the rail by pulling forward, backward and side to side. Rim-Mounted Tub Rail B was constructed of polypropylene with internal steel and coated aluminum components. Rim-Mounted Tub Rail B had a rubber-lined pressure-fit clamp, with adjustment of the clamp to the width of the bathtub achieved via a polypropylene dial and a polypropylene release lever. To install Rim-Mounted Tub Rail B, the user is instructed to clean and dry the bathtub, lift the release lever, rotate the dial to fit the bathtub rim, then lower the release lever to lock the Rim-Mounted Tub Rail in place. The user is instructed to check to make sure the installation is secure, but no specific instructions on how to perform this test are provided. None of the instructions for the devices tested indicated where the device should be installed. Each participant installed the Suction Cup Handhold and one of the two Rim-Mounted Tub Rails (randomly assigned), in randomized order.

### Environment

Data were collected in one session in a simulated bathroom environment (Figure 1). The lab is climate-controlled (ambient air temperature 21.86°C ± 0.92°C and humidity 25.86% ± 5.94% during data collection). A full-size enamel steel alcove bathtub (152.4 cm long, 76.2 cm wide, 41.3 cm tall; Cadet, Model # 0316RH, American Standard, Piscataway, USA) was mounted within an alcove. The plywood floor of the alcove was extended in front of the tub for participants to stand (152.4 cm by 61.0 cm). The walls of the alcove were constructed to meet Section 9.23 (Wood Frame Construction) of the National Building Code of Canada (National Research Council of Canada, 2020) with dimensional lumber and drywall panels, and covered by opaque white (3 mm) extruded acrylic sheeting, similar to that used to construct commercially-available bathtub and shower surrounds. In pilot testing, the research team successfully installed the Suction Cup Handholds to the surface. A continuous surface was selected rather than grouted tile, to allow more flexibility of handhold installation location, and greater potential for handhold installation success. A model shower head and faucet were installed at the end of the bathtub with the drain to provide an indication of the control end of the bathtub.

### Demographics

After consenting to participate in the study, participants completed a demographic survey to identify their gender, age, education level, self-reported language skills and technical skills (e.g., prior experience with home modifications or installing accessibility devices, including grab bars). The Activities-Specific Balance Confidence (ABC) scale was used to rate levels of self-confidence on their ability to maintain their balance while completing various activities (Myers et al., 1998; Powell & Myers, 1995). The Disabilities of the Arm, Shoulder and Hand (DASH) survey was used to assess functional capacity of the upper extremity (Angst et al., 2011). Participants were asked about their typical bathing practices and challenges while bathing, and how many times (if any) they had fallen while bathing.

Participant height (cm), weight (kg), and grip strength (kg, Jamar Hydraulic Hand Dynamometer, Sammons Preston Rolyan, Bolingbrook, USA) were measured. Researchers recorded two grip strength measurements for each arm and noted the participants’ dominant hand. The greater of the two grip strength measurements was selected as the representative measure.

### Usability Testing

Within the simulated bathtub environment, participants were provided with the following: one of the devices, the manual provided by the manufacturer, a chair, unscented bathtub cleaner, water, construction-style measuring tape, safety goggles, nitrile gloves, sponge, magnifying glass, knee pads, and a towel. All item recommended by the manufacturer (e.g., cleaning materials), if any, were included. Throughout the protocol, participants were recorded with two video cameras (HERO10, GoPro, San Mateo, USA) at different angles. Participants were given the following instructions:

*Please install the (handhold/tub rail) as if you were about to use it to enter the bathtub. Use the instructions provided in the box to guide you during the installation. You may use any materials in the bathroom to assist you during the installation tasks and you may read and reread the written instructions as many times as you feel is necessary. The researcher will not be able to assist you in any way, including answering any questions relating to how to install the (handhold/tub rail) or how to interpret the instructions. We ask that you complete the installation tasks to the best of your ability using the instructions and materials available in the bathroom. You may take a break at any time, but please inform the researcher when you are starting and stopping a break. Please also be reminded that you are free to stop participating and withdraw your consent at any time. When you are done installing the (handhold/tub rail), please say “done.”*

Participants were told that they would not receive intervention, verbal feedback or responses to questions during the installation task, except in the case of emergency.

Following installation of each device, participants completed a questionnaire about their experiences during the installation process. The questionnaires were composed of 7-point Likert scales to identify the comfort, stress, effort/fatigue, understanding of instructions, and confidence during installation of the handholds and tub rails. Participants also provided written comments. Participants were asked if they believed they had installed the device successfully.

All participants completed an exit questionnaire. Participants selected which device they would use in their homes based on their experiences installing and transferring (or imagining how they would use the device) with each model.

### Materials Testing

After the participant left the experimental environment, location and success of installation of each device was evaluated further by the research team. A diagram of the location of the handholds and tub rails was sketched, and distances (cm) from the floor, top-front edge of the bathtub rim, and control end of the bathtub were recorded. While participants were not explicitly told where to install the devices, we anticipated that the Suction Cup Handhold would be placed above the bathtub rim, between elbow and shoulder height, in alignment with local and international recommendations for the installation of handholds for bathtub entry and exit (CSA Group, 2018; Ontario Building Code, 2013).

The third author applied force to each of the devices using a digital force gage (microFET 2, Hoggan Scientific LLC, Salt Lake City, USA). For the Suction Cup Handhold, a strap attached the digital force gage to the handle, and a downwards force of 112.5 N was applied. For the Rim-Mounted Tub Rails, a downwards and lateral force of 280.5 N was applied. These values are consistent with the loading observed by Greene and colleagues (Greene et al., 2023) during an unperturbed bathtub exit using a vertical grab bar (8.9% body weight) and rim-mounted tub rail (22.2% body weight), respectively, scaled to a 95th percentile male (1263.5 N) (National Center for Health Statistics (CDC), 2015). The devices passed the loading test if no movement was observed when the specified load was applied. This method was selected over a true materials-testing approach with a mechanized load application as the method we used could be easily replicated by a clinician in a home environment.

### Data Analysis

Video recordings were coded by the first authors to identify total time required to install each device, number of transfers completed during installation (i.e., the number of times a participant moved both feet from inside the bathtub to outside the bathtub, or from outside the bathtub to inside the bathtub), and whether manufacturer instructions were followed for each device. Qualitative data (verbal and written responses) were summarized by the first authors.

### Statistics

Descriptive statistics were used to summarize participant demographics. Means and standard deviations were reported for continuous outcomes. The 7-point Likert scales and the number of successfully installed devices were summarized with counts.

## Findings

### Demographics

Twenty participants (10 men and 10 women) participated in the study (Table 1). Five men and five women installed each of the two Rim-Mounted Tub Rails. Participant demographics were similar to the older adult population in Canada (Statistics Canada, 2020), and all DASH and ABC scores were above the threshold for high functional status. Approximately half of the cohort (55%) reported challenges while bathing, 20% had previously fallen while bathing, and 35% currently used a grab bar during their typical bathing practice. Nearly half of participants had never attempted a home modification/renovation before, while 30% had attempted one, and 20% had completed several and were confident in their skills. The majority of participants (90%) were confident in their ability to install handholds and tub rails prior to completing the installation task.

**Table 1.** Participant Demographics.

	Suction Cup Handhold (*N* = 20)	Rim-Mounted Tub Rail A (*N* = 10)	Rim Mounted Tub Rail B (*N* = 10)
Gender	Count		
Men	10	5	5
Women	10	5	5
	Mean (*SD*)		
Age (years)	73.4 (5.7)	73.9 (5.6)	72.8 (5.9)
Height (cm)	169.7 (6.1)	170.1 (6.6)	169.3 (5.9)
Weight (kg)	72.5 (16.2)	72.3 (16.3)	72.7 (16.9)
Grip Strength (dominant hand, kg)	30.0 (10.0)	30.6 (10.0)	30.2 (10.7)
Grip Strength (non-dominant hand, kg)	28.3 (9.9)	28.0 (9.7)	28.8 (9.8)
DASH score	10.8 (9.3)	15.7 (10.6)	7.3 (6.8)
ABC score	94.7 (5.5)	96.2 (3.6)	93.2 (6.7)
	Count (%)		
Education level			
High School	2 (10)	2 (20)	0 (0)
Some college	7 (35)	3 (30)	4 (40)
Trade/technical/vocational	2 (10)	1 (10)	1 (10)
Bachelor’s degree	6 (30)	3 (20)	3 (30)
Graduate Degree	3 (15)	1 (10)	2 (20)
Current bathing practice			
Shower, standing	17 (85)	8 (80)	9 (90)
Shower, seated on a bath seat	1 (5)	0 (0)	1 (10)
Bath, seated on the floor	2 (10)	2 (20)	0 (0)
Reported challenges while bathing			
Balance (general)	3 (15)	3 (30)	0 (0)
Balance (while leaning over to wash feet)	2 (10)	2 (20)	0 (0)
Fatigue	1 (5)	1 (10)	0 (0)
Difficulty lifting foot over the bathtub rim	1 (5)	0 (0)	1 (10)
Dizziness	3 (15)	3 (30)	0 (0)
Avoiding slips	5 (25)	2 (20)	3 (30)
None of the above	9 (45)	4 (40)	5 (50)
Have fallen while bathing (number yes)	4 (20)	3 (30)	1 (10)
Currently have a grab bar in their bathroom that they typically use while bathing (number yes)	7 (35)	4 (40)	3 (30)
Experience with home modification/renovation			
I have never attempted	9 (45)	4 (40)	5 (50)
I have attempted, but do not have a lot of experience	6 (30)	3 (30)	3 (30)
I have completed several projects and am confident in my skills	4 (20)	2 (20)	2 (20)
No answer	1 (5)	1 (10)	0 (0)
Experience with accessibility device installation (number yes)	2 (10)	1 (10)	1 (10)
Confidence in having skills necessary to install a handhold/tub rail (number yes)	18 (90)	9 (90)	9 (90)

### Usability Testing

**Figure 2.**
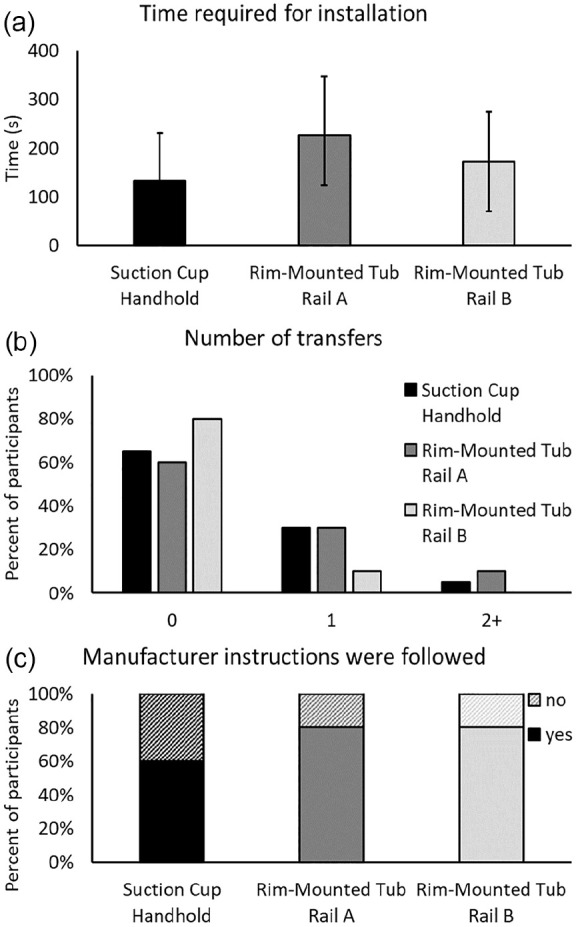
Characteristics of the installation process: (a) The time required to install the devices, from starting to read the instructions, to alerting the research team that the task was completed, (b) the number of transfers into and out of the bathtub during the installation process (one entry and one exit is one complete transfer), and (c) the percent of participants who followed all steps indicated in the manufacturer instructions.

Most handhold and tub rail installations took less than 4 min (Figure 2a). While only 10% of participants completed a transfer during installation of Rim-Mounted Tub Rail B, 35% of participants completed a transfer when installing the Suction Cup Handhold, and 40% completed a transfer when installing Rim-Mounted Tub Rail A (Figure 2b). Adherence to manufacturer instructions (i.e., completing every step, as described) was 60% for the Suction Cup Handhold, and 80% for each tub rail (Figure 2c). One quarter of participants did not clean the surface of the bathtub prior to installing the Suction Cup Handhold. The steps most frequently missed when installing the tub rails were cleaning the bathtub surface (15% of participants) and testing to ensure the grab bar was secured to the rim (20% of participants). One participant was unable to finish the installation task.

**Figure 3.**
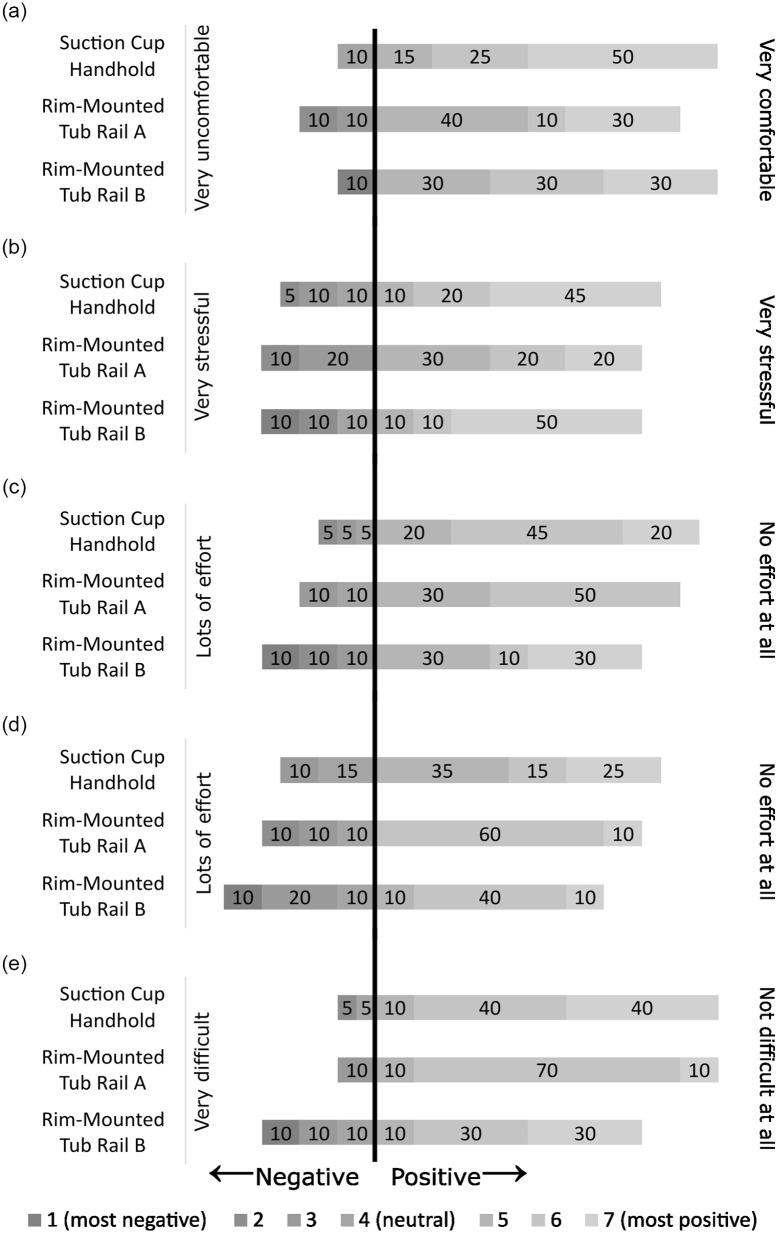
Participant ratings of experience during the installation of the devices. The depth of shade indicates scores that are negative (dark gray), neutral (mid gray), or positive (light gray). As well, ratings to the left of the vertical black bar are negative or neutral, while ratings to the right of the bar are positive. The width of the section of the bar for each rating indicates the proportion of the cohort that responded with that rating, and the number on each bar represents the percent of responses: (a) perceived comfort, (b) perceived stress, (c) perceived physical effort, (d) perceived mental effort, and (e) perceived overall difficulty.

Overall, participants rated the installation process positively for the Suction Cup Handhold and Rim-Mounted Tub Rail A, with 75% or more of participants rating the installation of these devices positively (score of 5, 6, or 7, or, e.g., “slightly comfortable,” “comfortable,” or “very comfortable”) on perceived comfort, stress, physical effort, mental effort and overall ease of installation (Figure 3). Rim-Mounted Tub Rail B was also rated well for perceived comfort, stress, and overall ease of installation (Figure 3a, b, and d), but 70% of participants reported that installation required a high physical effort (Figure 3c), while 40% reported that it required a high mental effort (Figure 3d). Regarding the Suction Cup Handhold, while participants overall commented that it was intuitive to use, one participant noted that it would be more challenging to install in a bathtub with tiles, and three participants said the location and position of installation was unclear. Several participants noted visual challenges when installing the Suction Cup Handhold, such as a small font on the manufacturer instructions (1 participant), or difficulty observing the red/green indicators (3 participants). Regarding Rim-Mounted Tub Rail A, one participant noted that it was substantially heavier than the Suction Cup Handhold, which they felt might be challenging if their strength was lower, and one participant noted that the locking collars used to adjust the height of the handle were challenging. Finally, four participants noted that the instructions for Rim-Mounted Tub Rail B were confusing.

**Figure 4.**
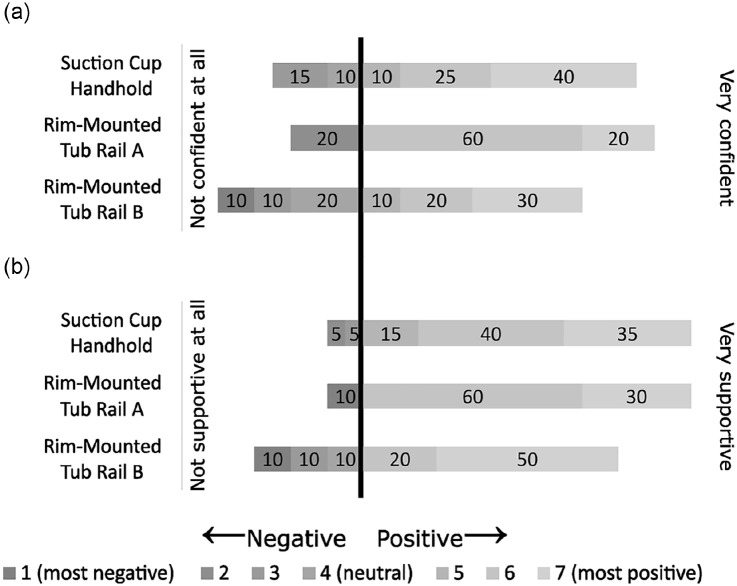
Participant rating of confidence in the installation of each device, and perceived support for bathing tasks. The depth of shade indicates scores that are negative (dark gray), neutral (mid gray), or positive (light gray). As well, ratings to the left of the vertical black bar are negative or neutral, while ratings to the right of the bar are positive. The width of the section of the bar for each rating indicates the proportion of the cohort that responded with that rating: (a) perceived confidence in installation and (b) perceived support for bathing tasks.

After installation, 75% of participants felt confident in their installation of the Suction Cup Handhold, 80% of participants felt confident in their installation of Rim-Mounted Tub Rail A, and 60% of participants felt confident with their installation of Rim-Mounted Tub Rail B (Figure 4). 90% of participants perceived that the Suction Cup Handhold and Rim-Mounted Tub Rail A would provide support for bathing tasks, while 70% of participants perceived that Rim-Mounted Tub Rail B would provide support for bathing tasks. Based on their overall experience with each device, when asked which device participants would use in their home, 60% of participants would install only a Suction Cup Handhold, 15% would install only a Rim-Mounted Tub Rail, and 25% would install both a Suction Cup Handhold and a Rim-Mounted Tub Rail in their home.

### Materials Testing

**Figure 5.**
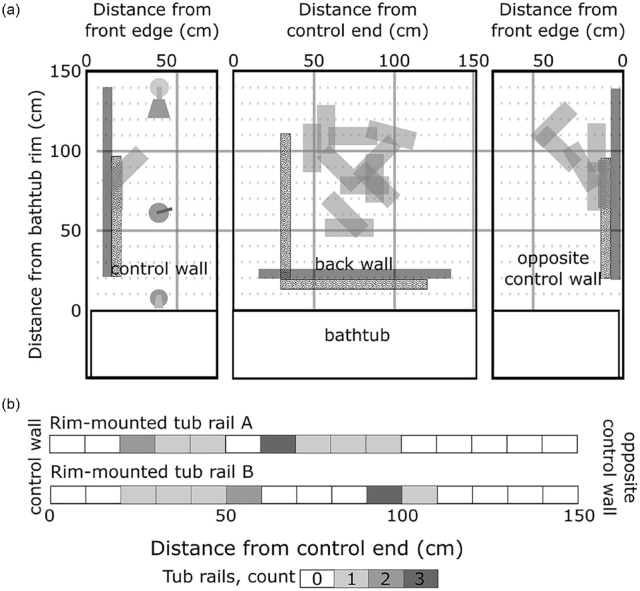
Placement locations of (a) Suction Cup Handholds and (b) Rim-Mounted Tub Rails. Participants commonly placed the Suction Cup Handholds (a) approximately 75 cm above the bathtub rim on the control wall or opposite the control wall, and 50 to 10 cm above the bathtub rim on the back wall. Handhold position and orientation are indicated with medium gray boxes. Location of grab bars for Barrier-Free bathtubs within the National Building Code are indicated with black boxes (corresponding to locations in Canadian Standards Association B651-12); location of grab bars for accessible bathtubs within the Ontario Building Code are indicated with dotted boxes. Placement of Rim-Mounted Tub Rails (b) are indicated as a distance between the control wall and opposite control wall, along the bathtub rim. Placements are blocked into 10 cm increments, with the number of installations at each location indicated by shading (see key, at bottom).

Placement of each device varied within our sample (Figure 5). Two participants placed the Suction Cup Handhold on the control wall, 11 participants placed the Suction Cup Handhold on the back wall, and six participants placed the Suction Cup Handhold opposite the control wall. One participant placed the Suction Cup Handhold face down on the bathtub rim, but removed it before it could be evaluated. Participants placed Rim-Mounted Tub Rail A a mean (standard deviation) 59.5 (27.8) cm from the control end of the bathtub, while they placed Rim-Mounted Tub Rail B 66.3 (30.9) cm from the control end of the bathtub. Three (15%) suction cup handhold installations, and 19 (95%) of Rim-Mounted Tub Rail installations failed mechanical load testing.

## Discussion

Our study explored whether older adults could install Suction Cup Handholds and Rim-Mounted Tub Rails according to manufacturer instructions to support bathing transfers, the ease with which they were able to do so, and the locations that they selected for each installation. While participants were confident that they could install these products, and generally confident that they had installed them correctly, no participant installed a Rim-Mounted Tub Rail effectively to support the anticipated loading during a bathing transfer. A higher success rate was observed with Suction Cup Handholds, with 85% of installations passing mechanical testing. Handhold location and orientation varied from expected placements to support bathtub entry and exit (CSA Group, 2018; Ontario Building Code, 2013). Only 40% of participants placed a handhold at the control end or opposite the control end, the anticipated location based on recommendations in existing codes and standards for entering and exiting the bathtub. Most participants were able to follow manufacturers’ instructions, complete the task quickly, and many participants rated the process as not physically or mentally challenging. However, one in three participants completed a transfer across the bathtub rim at least once before finishing the handhold installation, which puts them at risk for a fall.

Loading performance, relative to common applied loads, was good for the Suction Cup Handholds. Immediate mechanical testing indicated that the majority of the Suction Cup Handholds (85%) were able to withstand the 115N (consistent with the load applied to a vertical grab bar during bathtub exit with no perturbation or loss of balance) (Greene et al., 2023). However, it is important to note that in this study, participants were provided with a clean, smooth, grout-free acrylic wall. In a home scenario, the performance of these devices may differ, particularly in light of the finding that the installation step most frequently missed by participants was cleaning the tub/wall surface prior to handhold installation. Soap scum and mineral or dirt build-up would reduce the suction quality. Many bathtub and shower tiles are grouted, or have surface texture, porous material, or beveled edges. These factors interfere with suction and may make suction-cup handhold installation less feasible. We also performed the mechanical load testing directly after installation. Suction performance may decrease over time and with exposure to liquids, hygiene products, and changes in temperature and humidity. The performance of the Suction Cup Handholds in this study should be considered a best-case scenario, and may not reflect real-world performance.

While participants were confident in their installation of Rim-Mounted Tub Rails (70%), and believed the Rim-Mounted Tub Rails would be supportive of their bathing activities (80%), none of the Rim-Mounted Tub Rails withstood the 280 N force applied during load testing (consistent with the load applied to a rim mounted tub rail during bathtub exit with no perturbation or loss of balance) (Greene et al., 2023). Participants’ overall self-estimate of fall risk was low as reflected in their ABC scores. They may have interpreted, therefore, that the loading they would place on the tub rails would be very low, or that they would not use the device at all. It would be valuable to have anticipated loading performance, as well as clear methods for evaluating loading performance, included in product materials.

Similar to the Suction Cup Handholds, specific experimental conditions, such as the dimensions and texture of the bathtub rim, may have affected the performance of the Rim-Mounted Tub Rails. Even though approximately two-thirds of the length of the laboratory bathtub was un-curved and appeared to be a suitable match to the shape of the Rim-Mounted Tub Rail clamps, participants were not able to securely fix the clamp to the bathtub rim. While no participant successfully installed a Rim-Mounted Tub Rail on our study, these devices may perform better in bathtubs with other rim designs. However, Rim-Mounted Tub Rails would likely also perform poorly with bathtubs with sculpted or curved rims, decorative indents or shaping for features such as water jets, and bathtub rims that are too wide or narrow for the clamp. While we have no market data to evaluate how common the bathtub used in the current study is, the model is available from several popular home retailers in Canada, and is priced at the lower end of the range of bathtubs available on the market, and therefore likely represents a bathtub commonly installed in homes in Canada. Better specification of optimal and unacceptable use cases (e.g., surface materials) for temporary bathing assistive devices would be valuable.

While we anticipated devices would be placed in locations similar to those specified in codes and standards for permanent grab bars, the locations and orientations selected were varied. Canadian Standards Association (CSA) guidelines (CSA Group, 2018) recommends that two grab bars should be installed: one on the control wall in a vertical orientation (between 280 and 1,200 mm high), primarily for entering and exiting the bathtub; and one on the back wall in a horizontal orientation (180–280 mm above the rim), for support while bathing, and to get down to, and up from the bathtub floor. The Suction Cup Handhold was commonly (55%) placed on the back wall, above the height expected based on existing codes and standards. Despite the instruction to install the handle to support tub entry, some participants chose to place the grab bar in a location where it would support other bathing sub-tasks, such as hair washing, or because they perceived a need for greater stability when closing their eyes or tipping their head back. This identifies potential uses, and therefore handhold placement, that may not be explicitly considered in standard recommendations focused on entry or exit transfers. While for some the need for support during tasks beyond entry or exit may be addressed to some extent by a long vertical grab bar on the control wall, the relative consistency participants’ chosen installation locations in the middle of the back wall (an unusable location for entry or exit transfers) suggest that this location was perceived as particularly helpful by the older adults who participated in this study, suggesting that, for these participants, bathing tasks such as hair washing and tipping the head back with closed eyes may present a greater perceived risk than entry/exit transfers. While recognizing the potential merits of this placement, it must be noted that this location presents a safety concern if used for entry/exit transferring purposes as the user must lean over in order to reach it; greater trunk flexion is associated with poorer center of pressure control (King & Novak, 2017), and can increase the risk of falls (Grabiner et al., 2008; Owings et al., 2001). Grab bar placement and orientation in the context of task relevance is important for occupational therapists to consider when guiding clients on safe bathing practices; however, in the absence of direct occupational therapist guidance, the development of other resources would be valuable for directing users to appropriate grab bar configurations. Future research should examine the rationale behind grab bar placement and orientation to give greater insight into the desired use of grab bars.

The instructions provided by the manufacturers were limited in direct statements and objective values which could be used by the participant to determine whether the devices had been installed correctly. While the Suction Cup Handhold had color indicators to display when the device was attached correctly, it is unknown how well the indicator works on tiles of differing material or texture, or varying humidity conditions. Several participants noted that they were unable to determine from the indicator whether the handhold had been installed properly. The indicators may be too small to see clearly, or challenging to interpret for some users. Instructions for evaluating whether the installation of the rim-mounted tub rails were also vague and limited. None of the manufacturer instructions provided any directions regarding where to install the devices. While some guidance documents regarding grab bar locations do exist (CSA Group, 2018; Ontario Building Code, 2013), these are not designed for a lay audience. Development of knowledge translation and guidance strategies for the installation of, and evaluation of the installation of grab bars, such as pamphlets with suggested locations and objective testing techniques, or selection algorithms (Guay, Latulippe et al., 2020) may be helpful for improving installation success. The importance of clear and interpretable indicating tools can be extended to other assistive devices, such as brakes on walkers and wheelchairs. End users should be able to determine whether an assistive device is safe to use with minimal time or interpretation.

An overarching theme of this study was a mismatch between perceived risk and ability, and actual safety and performance. For example, participants completed transfers in order to install devices in 35% of cases. While there was no liquid in the bathtub, and participants were permitted to keep their shoes on, the transfer still carried a non-zero risk of slipping on the bathtub surface, or tripping or losing balance while crossing the bathtub rim. The risk of slipping would increase if the bathtub contained residual soapy water from a drained bath. Further, most participants thought they had installed a device effectively for the intended use, but most of the devices did not meet our loading criteria or anticipated positioning. The participants in this study did not differ substantially in demographics from participants in previous bathing transfer studies (Greene et al., 2023; Guay, Vinet et al., 2020; King & Novak, 2017; Nirmalanathan, 2019), so it would follow that the loads applied to the devices during non-falling transfers would be greater than estimated by the current cohort. The loading requirements would be greater still during loss of balance, and the optimal positioning may differ from where the devices in this study were installed (Nirmalanathan, 2019). Finally, even though 20% of participants had previously fallen while bathing, and nearly half of participants reported having bathing challenges for which a grab bar would be beneficial, and participants commonly commented that they felt grab bars were important safety devices for other people, but that they, themselves, did not need a grab bar. In older adults, perspectives on acceptance of use of assistive devices are complex, and strongly tied to identity as an assistive device user (Larsen et al., 2019). Greater understanding of self-perception of fall risk and need for assistive devices should be pursued through future research.

This study had several limitations. First, we evaluated only one bathtub, with one “ideal” wall surface, and three devices. Installation strategies and performance of these assistive devices may differ in actual home bathing environments. It may be valuable to evaluate installations in home bathrooms, with and without the guidance of occupational therapists. This would permit analysis of longitudinal performance under typical environmental and contaminant conditions. Second, the devices were re-used between participants. Participants were not required to complete steps such as removing the device from the packaging, or, in the case of Rim-Mounted Tub Rail B, initial assembly of separate components of the device, which could not be separated after assembly. These tasks may pose an additional challenge to users of these products. Third, given the relatively low sample size of the study and low frequency of effective device installations, we were unable to perform robust statistical analysis, which may have provided insight into what individual factors contributed toward installation outcomes. Fourth, we relied on self-reports of experience, physical balance, mobility, or cognitive ability of our participants, which may differ from actual ability.

## Conclusion

We aimed to determine whether older adults could install temporary handholds and tub rails to support their bathing needs. We found that the devices were easy for participants to install, and participants were confident in their installation, but the devices did not consistently meet loading criteria or anticipated positioning. Older adults who install handholds or tub rails in their home without the assistance of an occupational therapist may require guidance tools for determining where and how to install these devices, and how to determine whether it meets their needs.
